# Self-Healing and Mechanical Properties of Thermoplastic Polyurethane/Eugenol-Based Phenoxy Resin Blends via Exchange Reactions

**DOI:** 10.3390/polym12051011

**Published:** 2020-04-28

**Authors:** Jing-Yu Liang, Se-Ra Shin, Soo-Hyoung Lee, Dai-Soo Lee

**Affiliations:** 1 Department of Semiconductor and Chemical Engineering, Chonbuk National University, 567 Baekje-daero, Deokjin-gu, Jeonju 54896, Korea; liangjy@naver.com; 2 Research Institute of Jungwoo Fine Chemical, 68-3 Seogam-ro 1-gil, Iksan 54586, Korea; srshin89@jbnu.ac.kr; 3 Research Institute of Future Energy, Chonbuk National University, 567 Baekje-daero, Deokjini-gu, Jeonju 54896, Korea

**Keywords:** TPU, phenoxy resin, blend, exchange reaction, self-healing

## Abstract

The possibility of exchange reactions and thermal self-healing in blends of thermoplastic polyurethane (TPU) and phenoxy resin was investigated herein. The analyses were based on characterization obtained via differential scanning calorimetry (DSC), Fourier transform infrared spectroscopy (FTIR), dynamic mechanical analysis (DMA), and tensile test. A new phenoxy resin was synthesized from eugenol, and blends with different types of TPU were prepared to investigate the exchange reaction, thermal self-healing, and mechanical properties. The influence of phenoxy resin content on the mechanical behavior and healing efficiency was studied. Improvement of storage modulus owing to the increase of phenoxy resin content was observed. Results suggest that the exchange reaction between phenoxy- and ester-type TPU occurred during thermal treatment. However, little exchange occurred between phenoxy resin and ether-type TPU. Specifically, only ester-type TPU exhibited a significant exchange reaction in the phenoxy resin blend. Furthermore, in the presence of a catalyst (e.g., zinc acetate), the exchange reaction readily occurred, and the healing efficiency improved by the addition of the catalyst and increase in the phenoxy content.

## 1. Introduction

Polymer blends have received considerable attention from both the industrial and scientific communities because they are economical materials that can be used to create new compounds in comparison with copolymers prepared from polymerizations [1,2,3]. The miscibility of different polymers for the preparation of blends has been extensively studied. However, many polymer blends are immiscible and require a compatibilization process to improve the performances of blends. Frequently, reactive polymer blends based on the functional groups such as carboxylic acid, amine, and hydroxyl groups were studied for the efficient compatibilizations of immiscible polymers [4,5,6].

Phenoxy resin is an amorphous thermoplastic, which has been extensively used in many fields such as in adhesives, coatings, and engineering plastics. However, there are limitations in the application of these resins due to brittleness and easy stress cracking [7,8]. The hydroxyl group of phenoxy resin is an active group and can easily react with proton-accepting functional groups in the polymers. Therefore, to improve the physicochemical properties, phenoxy resin is frequently used in polymer blends to modify polymeric compounds [9]. Phenoxy blends have been studied by several researchers. Dixit and his coworkers used phenoxy resin to modify epoxy blends [10]. Eguiazabal investigated the interchange reactions and miscibilities of the poly (butylene terephthalate) (PBT)/phenoxy blends and the poly (ethylene terephthalate) (PET)/phenoxy blends based on the thermal properties of the blends [11,12]. Goh et al. studied the miscibility behavior of various phenoxy/polymethacrylate blends [13]. Other phenoxy blends are also available that undergo transreaction or exchange reaction during mixing at high temperature such as poly (1,4-butylene adipate)/phenoxy [9], PBT/phenoxy [14], and poly (trimethylene terephthalate)/phenoxy blends [15].

Polyurethane (PU) is one of versatile polymers for foams, elastomers, fibers, coating, and adhesive. PUs are generally manufactured from polyols and polyisocyanates for various applications. Thermoplastic polyurethanes (TPUs), one of representative thermoplastic elastomers, possess good mechanical properties such as high elasticity, low temperature flexibility, and abrasion resistance [16]. TPUs also exhibit preferable compatibility with phenoxy resin [17,18], and they have been blended to prepare adhesives. The linear structure of TPU is prepared from three main components (i.e., long chain diols (polyester or polyether based), diisocyanates (aromatic or aliphatic types), and short chain diols), which form hard and soft segments [19]. Ester-type TPU, which contains ester and urethane groups, can be blended phenoxy resin and compatibilized through transreaction or an exchange reaction, which forms copolymers and a crosslinked structure [20,21]. Blends are convenient engineering materials because they possess the advantages of each component. The degree of compatibility is a factor that determines the final properties of the blend, and other factors include chemical composition, molecular weight, and catalysts [22,23]. A compatible polymer blend exhibits mechanical properties proportional to the ratio of the constituents of the blend [24]. 

Eugenol, 4-allyl-2-methoxyphenol, is a relatively cheap bio-based compound with multifunctional groups. It is widely used in antioxidants, food, perfumes, and drugs [25,26]. Considering its potential abundant production and unique structure which includes methoxy-substituted phenolic ring and allyl group, it has been regarded as an ideal candidate to replace petroleum-based compounds. A variety of polymers have been prepared from eugenol based monomers [27,28,29,30,31,32,33,34]. Deng used eugenol as a raw material to prepare oil-absorbing microspheres by suspension polymerization technique [30]. Liu prepared high bio-content thermosetting polymers by using free radical copolymerization of methacrylated eugenol and acrylated epoxidized soybean oil [31]. Thirukumaran [25] reported the synthesis of benzoxazine-based phenolic resin using eugenol, and studied the effect of incorporation of eugenol in benzoxazine-based resins on the thermal properties. Zhang [32] synthesized renewable methacrylated eugenol (ME) from eugenol, which was effective reactive diluent to replace styrene for commercially available resin. 

Herein, the interactions between TPU and phenoxy resin in blends of TPU/phenoxy resin were studied. Specifically, thermal self-healing and mechanical properties resulting from the exchange reactions of phenoxy resin and TPUs were investigated. To the best of our knowledge, no reports are available on the self-healing of TPU/phenoxy resins at elevated temperatures. In this article, we report the preparation of phenoxy resin from eugenol. Eugenol epoxy (EE) was synthesized and phenoxy resin was obtained by polymerization of EE. The TPU/phenoxy resin blends were prepared with or without catalysts. The ratio of the constituents of the blend and catalyst were varied, and the effects on the thermal, mechanical, and self-healing properties were reported.

## 2. Materials and Methods 

### 2.1. Materials

**Scheme 1:**
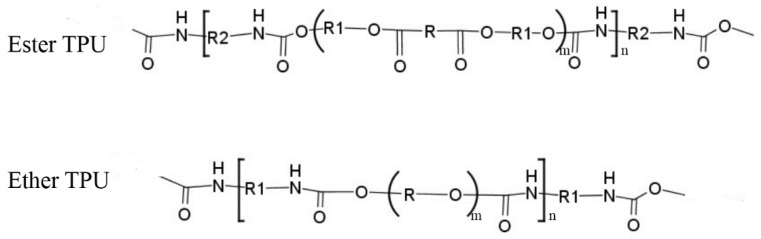
Repeat structures of ester and ether thermoplastic polyurethanes (TPUs).

Eugenol (99%), 3-chloroperoxybenzoic (m-CPBA, <77%), 1-methylimidazole (99%), zinc acetate (99.9%), 1,5,7-triazabicyclo[4,4,0]dec-5-ene (TBD, 98%), and dibutyltin dilaurate (DBTDL, 99%) were purchased from Sigma-Aldrich Chemical (Yongin City, South Korea). Ester-type TPU (Neothane 5285 AP) was obtained from Dong Sung Corporation (Busan, South Korea), and ether-type TPU (Desmopan 9395AU) was purchased from Covestro Deutschland AG (Leverkusen, Germany). Average molecular weights of TPUs determined by Gel Permeation Chromatography (GPC) are given in Table S1. Sodium thiosulfate (Na_2_S_2_O_3_, 98%), sodium bicarbonate (NaHCO_3_, 99%), sodium chloride (NaCl, 99%), and anhydrous sodium sulfate (Na_2_SO_4_, 99%) were purchased from Aldrich Chemical (Yongin City, South Korea). The solvents used in this work, including dichloromethane (CH_2_Cl_2_, 99%) and dimethylformamide (DMF, 99%), were supplied by Samchun Pure Chemical (Pyeongtaek-si, South Korea). All materials were used without further purification. The typical structures of TPUs are shown in Scheme 1.

### 2.2. Synthesis of TPU/Phenoxy Blends

#### 2.2.1. Synthesis of EE

**Scheme 2:**
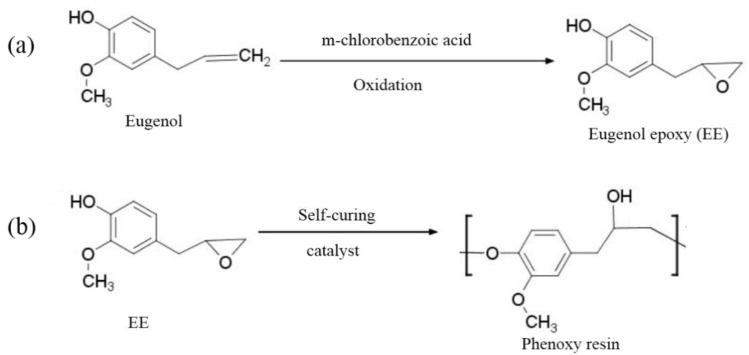
Synthesis route and structure of (**a**) eugenol epoxy (EE) and (**b**) phenoxy resin.

m-CPBA (206 g) was dissolved in 1000 mL of anhydrous dichloromethane in a 2000-mL round bottomed flask. Eugenol (66 g) was dissolved in 500 mL of anhydrous dichloromethane and slowly added to the m-CPBA solution over 1 h. The reaction mixture was stirred at room temperature for 24 h. The solution was filtered through a funnel, then washed with sodium thiosulfate solution (Na_2_S_2_O_3_) and solution of sodium bicarbonate (NaHCO_3_). The organic layer was dried under Na_2_SO_4_ and evaporated under vacuum. EE was obtained in 47% yield, which is similar to a 45% yield reported in a previous study [35]. The corresponding preparation route and structure are shown in Scheme 2a.

#### 2.2.2. Synthesis of Phenoxy Resin from EE

In this experiment, EE undergoes self-polymerization in the presence of a catalyst, 1-methylimidazole (1 wt % on the basis of the total weight of EE), by the reaction between epoxy group and aromatic hydroxyl group. The EE and the catalyst mixture were maintained at room temperature for 30 min to achieve good mixing. Then, the mixture was transferred into a mold and maintained at 110 °C for 24 h. The polymerized sample was used as the phenoxy resin and its corresponding structure is shown in Scheme 2b. Average molecular weights of the phenoxy resin determined by GPC are given in Table S1.

#### 2.2.3. Preparation of TPU/Phenoxy Resin Blends

The TPU/phenoxy blend samples were prepared via solution blending using DMF as a cosolvent, the weight ratio of blend/DMF was 1/6. The polymer solutions were thoroughly stirred and then cast at 100 °C as films on Teflon^®^ molds of 200 × 200 mm, and the thickness of films after drying was about 1.5 mm. The solvent in the solutions was allowed to evaporate at 100 °C. Then, degassing was performed for 2 weeks in a vacuum oven at 110 °C to remove residual solvent from the samples. Ester- and ether-type TPUs were used to study the mechanical properties of the blends. The TPU/phenoxy compositions were 100/0, 90/10, and 80/20. Zinc acetate, DBTDL, and TBD were used as catalysts to modify the abovementioned blends, respectively. Table 1 lists the sample code and compositions of the blends.

### 2.3. Characterization

FT-IR spectra were obtained employing FT-IR-302 from Jasco (Tokyo, Japan). The samples were smeared on a KBr window and scanned from 500 to 4000 cm^−1^. Differential scanning calorimetry was performed using a Q20 DSC from TA instrument (New Castle, DE, USA). The DSC measurements were performed at a heating rate of 10 min^−1^ from −80 to 200 °C. ^1^H-NMR measurements were performed using a 600 MHz spectrometer (JNMECA600, JEOL, Tokyo, Japan). Tensile properties and self-healing efficiency were measured using a universal testing machine (UTM, LR5K Plus from LLOYD, West Sussex, UK). The measurements were conducted with 4 specimens per sample at 25 °C with a cross-head speed of 500 mm min^−1^ following the ASTM-D638 method, and the dimensions of the dog bone test samples were 20 × 5 × 1.5 mm. To evaluate the self-healing efficiency of the materials, the specimens (with the abovementioned dimensions) were cut into two parts in the middle of the gauge length, and the two cut surfaces were brought back in contact manually as shown in Scheme S1. The samples were allowed to heal for 24 h at 150 °C, and was subsequently tested on a universal testing machine. The healing efficiency was obtained from cut and heal tests in tensile property measurements with the following equation:

Healing efficiency = (σ_healing_/σ_original_) × 100% (1)

where σ_healing_ and σ_original_ are tensile strength of healed specimen and original specimen respectively, the schematics of self-healing process was given in supporting information Scheme S1. Dynamic mechanical analysis (DMA) was conducted using a Q800 from TA instrument (New Castle, DE, USA). The dimensions of the samples were 16 × 5 × 1.5 mm and the heating rate and temperature range were 5 °C min^−1^ and −100–200 °C, respectively; and the frequency was 1 Hz.

## 3. Results and Discussion

### 3.1. Synthesis and Characterization of Phenoxy Resin

**Figure 1:**
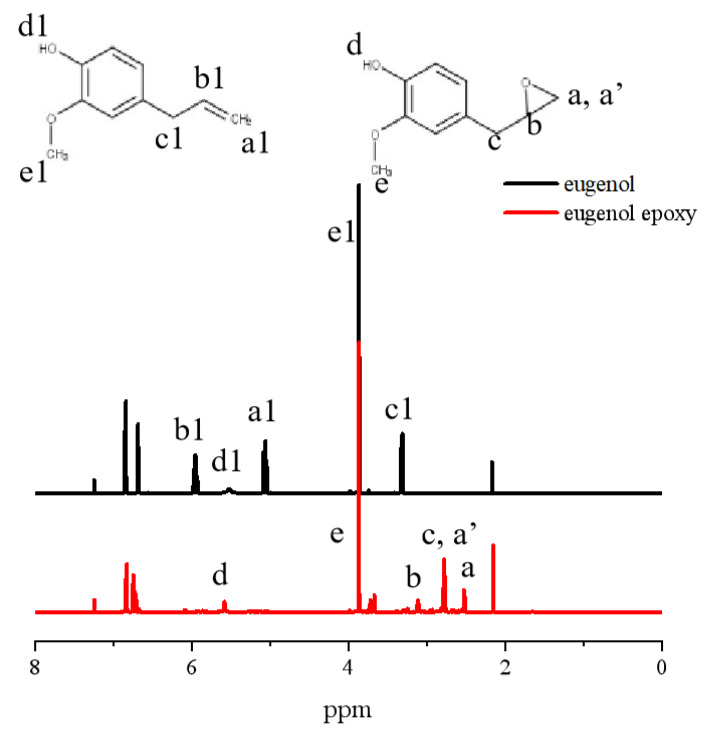
^1^H NMR spectra of eugenol and EE, respectively.

As shown in Scheme 2a, EE was synthesized from eugenol and 3-chloroperoxybenzoic acid (as an oxidizing agent) at room temperature. EE was obtained by filtering and washing after the synthesis, and was characterized. The FTIR spectra given in Figure S1a show that eugenol exhibited an allyl C=C stretching vibration absorption peak at 1640 cm^−1^, and this C=C peak disappeared in the FTIR spectrum of EE. The absorption of the C-O-C vibration band of the epoxide group in EE is observed at 850 cm^−1^, which is not observed in eugenol. This suggests that the allyl group was converted into the epoxide group. ^1^H NMR spectra of eugenol and EE are shown in Figure 1. The chemical structure of EE was confirmed by the appearance of new signals (a’), (c, a), (b) at 2.53, 2.79, and 3.12 ppm (which correspond to the protons of the epoxide groups), and the disappearance of the signals of (b1) and (a1, a1’) at 6.1 and 5.1 ppm, respectively, which are assigned to the protons of allyl groups. The ^1^H NMR and FTIR spectra indicate that EE was successfully synthesized. The ^1^H NMR spectrum of the phenoxy resin is given in Figure S1b. Successful polymerization of EE into the phenoxy resin was confirmed by the disappearance of the signal at 5.7 ppm corresponding to the protons of aromatic hydroxyl groups and the appearance of new signal at 2.6 ppm corresponding to the protons of the new hydroxyl groups.

**Figure 2:**
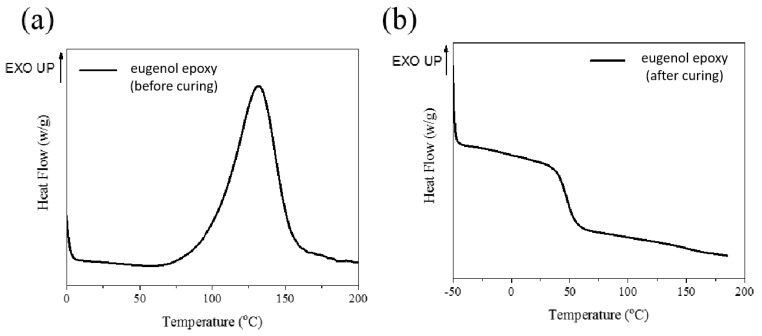
Differential scanning calorimetry (DSC) thermograms of EE before curing (**a**) and after curing (**b**).

The exothermic polymerization reaction of EE in the presence of the catalyst, 1-methylimidazole, and the thermal properties of the obtained polymer are shown in Figure 2. Figure 2a shows the DSC thermogram, which reveals that exothermal reaction heats occurred at a heating rate of 10 °C/min in the temperature range of 50 °C–200 °C. The temperature scan showed one exothermic peak with the onset temperature at 90 °C; the peak temperature was at 131 °C; the heat of reaction was 74 kJ/mol. In the second scan shown in Figure 2b, no additional exothermic peaks were observed, which confirms that the polymerization reaction was completed. The T_g_ value of phenoxy resin was 40 °C. DSC data confirm that EE undergoes polymerization in the presence of the catalyst.

### 3.2. Thermal Properties of Blends

**Figure 3:**
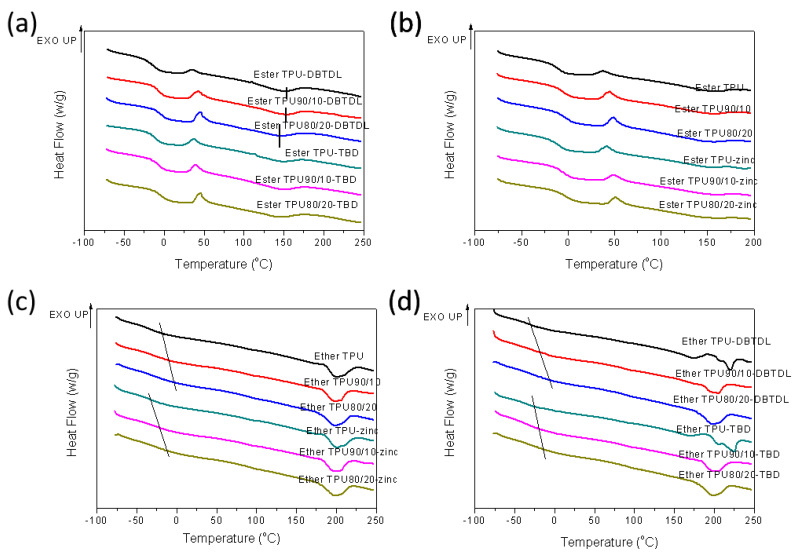
DSC curves for the TPU/phenoxy blends: (**a**,**b**) Ester TPU/phenoxy blends with different catalysts; (**c**,**d**) Ether TPU/phenoxy with different catalysts.

DSC thermpgrams of the TPU/phenoxy resin blends are shown in Figure 3. Enthalpy changes due to the glass-rubbery transition temperatures of soft segment in TPUs, T_gs_’s, were observed at −25–−7 °C and endotherms due to the hard segment melting temperatures of TPUs, T_m_’s, appeared 139–200 °C. Ester-type TPU showed exotherms at 35–49 °C due to the crystallization temperatures, T_c_’s, of soft segments in Figure 3a,b. Thermal transition temperatures of TPU/phenoxy resin blends are summarized in Table 2. Contents of eugenol based phenoxy resin in the blends affected the T_gs_ of the TPU. Specifically, with increasing the phenoxy content of the blends, T_gs_’s of TPU increased while T_m_ decreased independent of catalyst types, implying the dissolution of phenoxy resin into the soft segment domain and increase of phase mixing of soft and hard segments of TPU. It seems that the hard segments of TPU dissolved in the soft segment domains owing to the increase of phase mixing by the exchange reaction with phenoxy resin described below in Figure 3 [36]. Figure 3c,d shows that the ether-type TPU blends have lower T_gs_’s and higher T_m_’s compared with the ester-type TPU blends, and the ether-type TPU blends do not show exotherms due to the crystallization of soft segments. Similar to the ester-type TPU/phenoxy blends, T_gs_’s of TPU increased while T_m_ decreased with the increasing content of phenoxy resin in the blends. Compared with previous reports on eugenol based polymers, the TPU blends showed relatively low glass transition temperatures due to the microphase separation of segmented TPUs [33,36]. Enthalpy changes due to the exchange reactions at elevated temperatures were not observed in DSC, being attributable to little change in the type of chemical bonds through the exchange reactions. 

The thermal stability of the blends was investigated employing TGA. Figure S2 shows TGA thermograms of ester-type TPU/phenoxy resin blends under nitrogen atmosphere. Thermal degradation temperature for 5% weight loss (T_d 5%_) decreased with increasing content of phenoxy resin. However, in the presence of zinc acetate as a catalyst for the exchange reactions, T_d 5%_, the measure of thermal stability of blends, slightly increased with increase the phenoxy resin content in the blend. It was also observed the char content at 700 °C was increased with increasing phenoxy content in the presence of zinc acetate. It is speculated that the exchange reactions of the phenoxy resin and TPU in the blends improved the thermal stability due to the aromatic moiety derived from eugenol. 

### 3.3. Mechanical Properties and Exchange Reaction of the Blends

**Figure 4:**
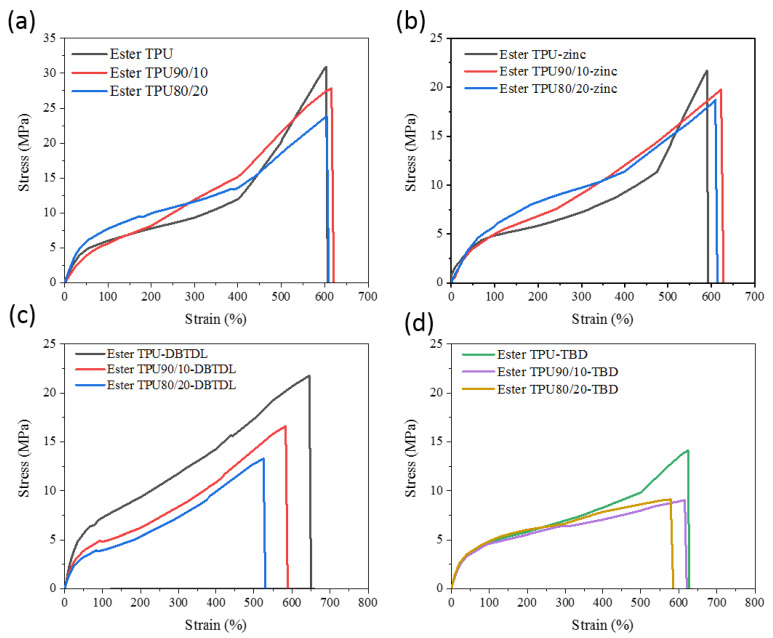
Stress-strain curves for the ester TPU/phenoxy resin blends: (**a**) ester TPU/phenoxy without the catalyst; (**b**) ester TPU/phenoxy with zinc acetate as the catalyst; (**c**) ester TPU/phenoxy with DBTDL as the catalyst; (**d**) ester TPU/phenoxy with TBD as the catalyst.

**Figure 5:**
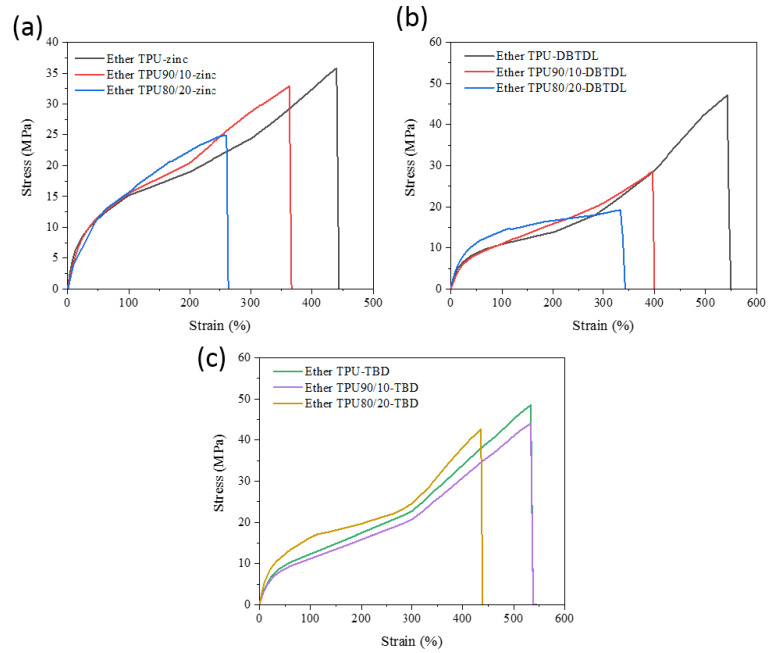
Stress-strain curves for the ether TPU/phenoxy resin blends: (**a**) ether TPU/phenoxy with zinc acetate as the catalyst; (**b**) ether TPU/phenoxy with DBTDL as the catalyst; (**c**) ether TPU/phenoxy with TBD as the catalyst.

Figure 4 and Figure 5 show the representative stress–strain curves of the TPU/phenoxy blends and the results are summarized in Table 2. It was observed that initial slopes, elastic moduli, changed little while stress at large deformation were affected largely by the composition of the blends. In general, intermolecular interactions such as hydrogen bonding affect tensile properties at large deformations [37]. The tensile strength and elongation at break of TPU/phenoxy blends decreased with increasing the phenoxy content due to the inherent low strength and low elongation of the phenoxy resin in comparison with TPUs. Figure 4a shows that the addition of the catalyst into the TPU/phenoxy blends resulted in the decrease of the mechanical properties compared with those without the catalyst. The catalyst considerably affects tensile strength. It was found that tensile stress significantly decreased when TBD was used as the catalyst; zinc acetate had a lower effect on the tensile stress. Figure 5 shows that the ether-type TPU blends with different amounts of phenoxy resin; their tensile strengths are higher compared with those of ester-type TPU. In addition, with increasing the phenoxy content, the tensile strengths decreased. The stress-strain curves indicate that phenoxy resin content and catalysts considerably affected the mechanical properties. 

**Figure 6:**
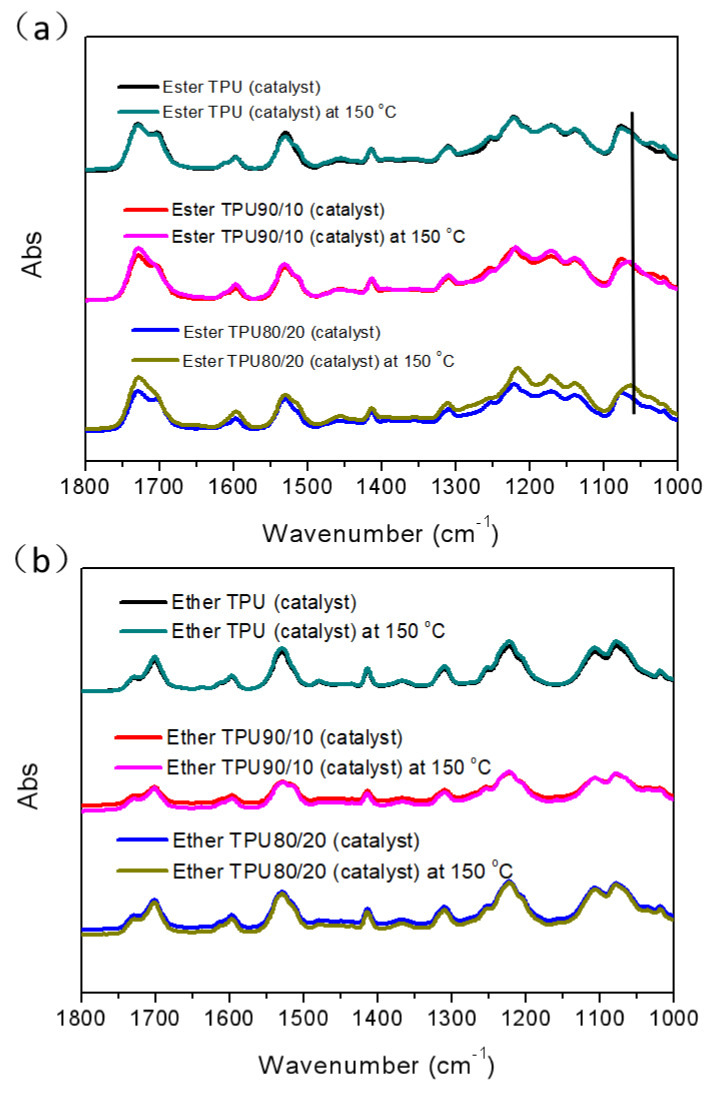
ATR-FTIR spectra of heat-treated TPU/phenoxy blends at low wavenumbers: (**a**) Ester TPU/phenoxy with the catalyst; (**b**) ether TPU/phenoxy with the catalyst.

**Figure 7:**
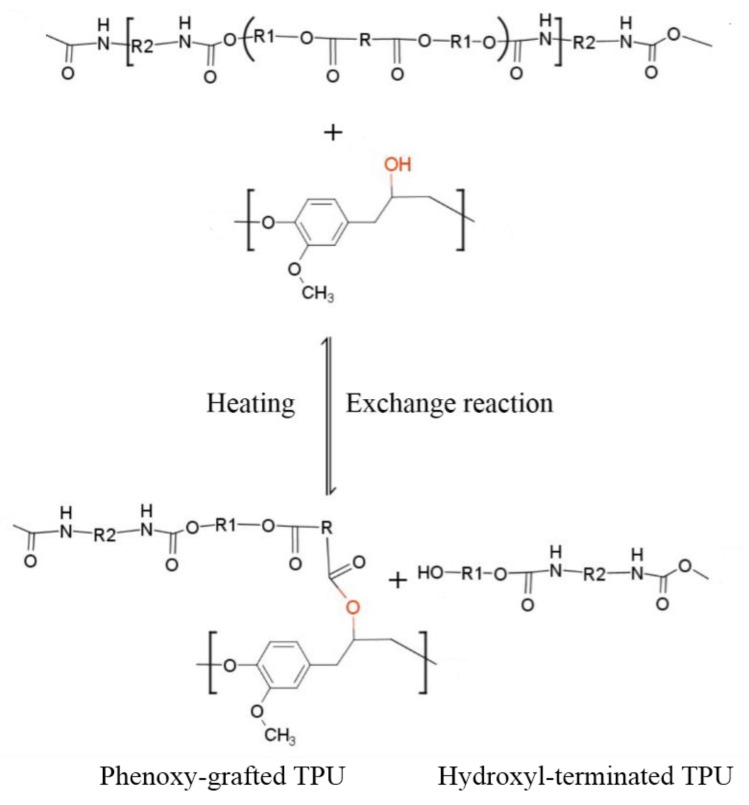
Schematic of the exchange reaction between ester TPU and phenoxy resin.

To clearly understand the exchange reaction in the TPU/phenoxy blends, FTIR analysis was used to identify the species responsible for the chemical interactions in TPU/phenoxy blends [38]. The ATR-FTIR spectra were acquired before and after heat treatment at 150 °C for 24 h. Figure 6 shows that exchange reaction may have occurred after the heat treatment of the ester-type TPU/phenoxy blends. Spectral changes occurred in the 1800 to 1000 cm^−1^ region owing to the change in alcoholic C-O stretching bonds during the exchange reaction. Figure 6a shows the effect of phenoxy on the exchange reaction. Neat ester-type TPU showed little change in the peak at 1090 and 1730 cm^−1^. For the ester TPU/phenoxy blends, the absorption peak at 1080 cm^−1^ corresponds to the C-O bond of TPU, the absorption peak at 1700 cm^−1^ corresponds to the hydrogen-bonded carbonyl group while the absorption peak at 1730 cm^−1^ corresponds to the non-bonded carbonyl group of TPUs. Following the heat treatment, the peak of C-O bond shifts to a lower wavenumber, and the peak intensity of non-bonded carbonyl bond at 1730 cm^−1^ became strong relatively due to the decrease of hydrogen bonded carbonyl through the exchange reaction between TPU and hydroxyl group. The peak of C-O at 1063 cm^−1^ corresponds to the increase of primary hydroxyl group [39], which indicates that an exchange reaction occurred in the TPU/phenoxy blends. Figure 6b shows that there are no exchange reactions in the ether-type TPU blends even after adding phenoxy because there is no change in the C-O group by the post heat treatment. Thus, exchange reactions occur more easily in ester groups compared with urethane groups, and the exchange reactions occur significantly in ester-type TPU/phenoxy blends. The spectroscopic features are attributed to the thermal exchange reaction between the hydroxyl group of the phenoxy resin and ester groups, as shown in Figure 7.

### 3.4. Dynamic Mechanical Properties of the Blends

**Figure 8:**
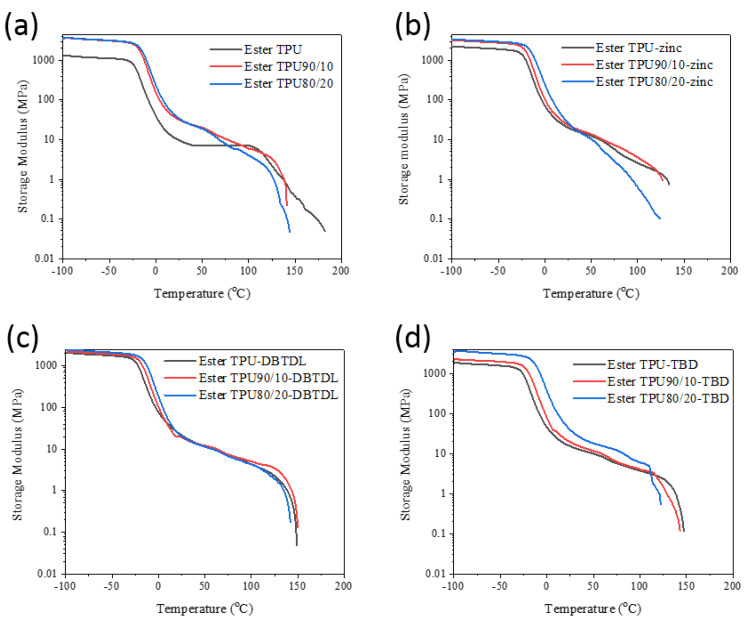
Dynamic mechanical analysis (DMA) curves of ester TPU/phenoxy blends: (**a**) Storage modulus for the ester TPU blends without the catalyst; (**b**) storage modulus for the ester TPU blends with zinc acetate as the catalyst; (**c**) storage modulus for the ester TPU blends with DBTDL as the catalyst; (**d**) storage modulus for the ester TPU blends with TBD as the catalyst.

Figure 8 shows the temperature-dependent dynamic mechanical properties of ester TPU/phenoxy blends. Specifically, Figure 8a–d shows the storage moduli of the TPU blends with different catalysts. Table 2 gives the storage moduli for the TPU/phenoxy blends. Storage moduli may be used to determine elastic properties. Figure 8 shows that when the temperature was below the glass transition temperature of the soft segment domains, a high storage modulus was obtained, and neat TPU has the lowest storage modulus compared with those of the TPU blends. When the phenoxy content was increased, the storage modulus increased in the glassy region. In general, the storage moduli of TPUs depend on the soft segment concentration, the lower is the soft segment concentration, the higher is the storage modulus. It is postulated that the phenoxy resin in the blends underwent dissolution into the continuous soft segment domain in TPU. Thus, all TPU blends exhibit increased storage moduli at temperatures lower than that of the soft segment T_gs_. Above T_gs_, the storage modulus decreased with increasing temperature owing to glass-rubbery transition of the soft segment domain. Figure S3 shows the storage moduli of ether TPU/phenoxy blends. The trend is similar to that of ester-type TPU blends. Specifically, with increasing phenoxy content, the storage modulus increased. Furthermore, ether-type TPU blends exhibited higher storage moduli compared with those of ester-type TPU blends. The catalyst did not considerably affect the storage modulus. The addition of the catalyst increased the storage modulus of neat TPU, but decreased the same for TPU/phenoxy blends.

Figures S4 and S5 show the value of tan delta of TPU/phenoxy blends. The glass transition temperatures of the blends were determined from the peak maxima of tan delta. All samples exhibited a T_gs_ of approximately 0 °C, and the peak shifted to higher temperatures with increasing phenoxy content. The peak did not considerably change with different catalysts. This suggests that the motion of TPU molecular chains was effectively restricted by phenoxy.

### 3.5. Self-Healing and Mechanical Properties

**Figure 9:**
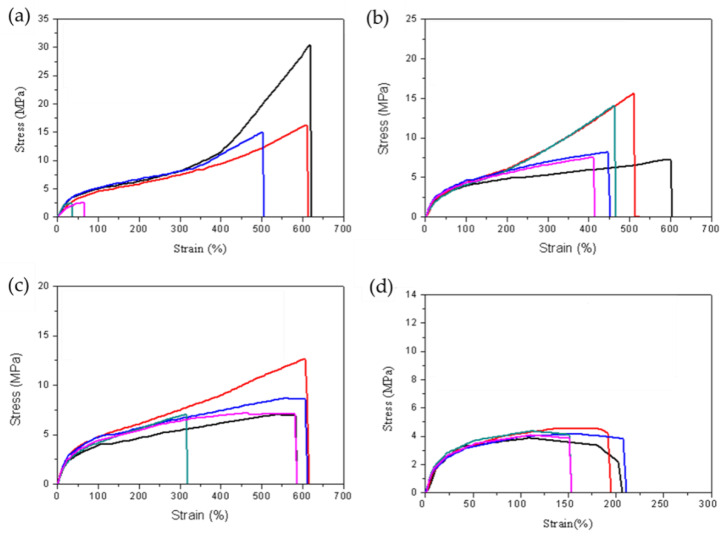
Self-healing properties of ester TPU/phenoxy blends: (**a**) blends without the catalyst; (**b**) blends with zinc acetate as the catalyst; (**c**) blends with DBTDL as the catalyst; (**d**) blends with TBD as the catalyst. Colored lines represent samples of different composition and thermal history: (━) Pristine ester-type TPU; (━) Ester TPU90/10 after heat treatment at 150 °C; (━) Ester TPU80/20 after heat treatment at 150 °C; (━) Ester TPU90/10 after cut and heal at 150 °C; (━) Ester TPU90/20 after cut and heal at 150 °C.

**Figure 10:**
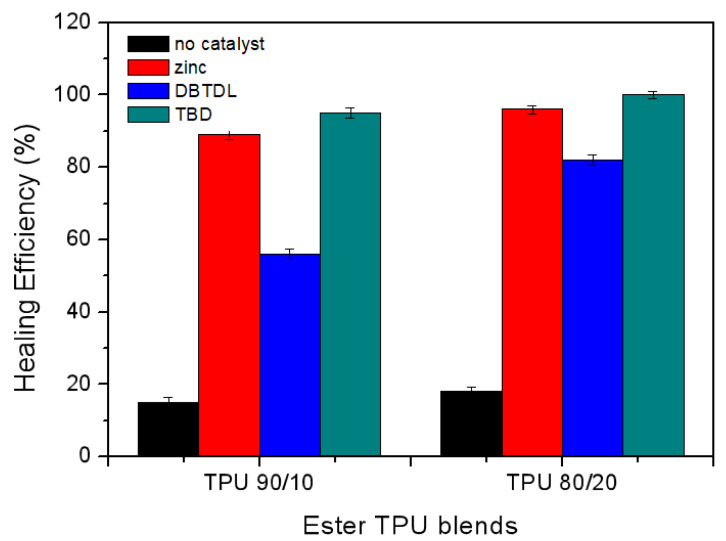
Healing efficiency of ester TPU/phenoxy blends with different catalysts.

To investigate the exchange reaction and the self-healing properties of TPU/phenoxy blends, the cut and heal tests of the tensile properties for the blends were carried out. Both ether and ester TPUs of neat polymers didn’t show thermal healing properties at 150 °C in this study, even in the presence of catalysts. It was found that ester TPU/phenoxy resin blends showed thermal healing in cut and heal tests at 150 °C while ether TPU/phenoxy resin blends did not. The ether TPU/phenoxy blends did not exhibit self-healing properties because the exchange reaction hardly occurred at 150 °C as shown in Figure 6b. Figure 9 shows the tensile properties of ester-type TPU/phenoxy resin blends after healing by the heat treatment at 150 °C. In order to preclude the thermal history effects of the samples in the studies by the cut and heal tests, control samples without cut were also treated at the same thermal conditions. In Table 3, results of cut and heal tests are summarized. It is of interest to note that the thermal healing efficiencies of the ester TPU/phenoxy resin blends increased with increasing the phenoxy resin contents due to more exchange reactions in the blend. Figure 10 shows the healing efficiency of the TPU blends with different catalysts. It was found that the healing efficiency were improved considerably by the catalysts. The catalysts differently affected the healing properties. Results indicate that the catalyst positively affected the exchange reaction between ester-type TPU and phenoxy resin. It was observed that the healing efficiency of the blends with TBD was the highest among the blends. However, the tensile strength of the blends was decreased very much after the heat treatment due to the change of the microstructure of the polymer chains via the exchange reactions between TPU and phenoxy resin and TBD is not favored practically. Ester TPU/phenoxy resin blends with zinc acetate as the catalyst also resulted in excellent healing efficiencies, maintaining the tensile properties reasonably. 

## 4. Conclusions

The self-healing and exchange reactions in the blends of TPU and phenoxy resin were investigated in this study. Eugenol as a biomass was used to synthesize EE from which the phenoxy resin was obtained by ring opening polymerization. Ester and ether-type TPU/phenoxy blends were prepared by solution casting. It was observed that exchange reactions occurred between urethane, ester, and hydroxyl groups of ester-type TPU/phenoxy resin blends during heat treatment at 150 °C while exchange reactions hardly occurred in ether type TPU/phenoxy blends. It is speculated that the hydroxyl group in the repeating unit of phenoxy underwent exchange reactions efficiently with ester- type TPU in the blends. This reaction imparted blends thermal self-healing properties to the ester-type TPU/phenoxy blends. Especially, in the presence of catalyst for the exchange reactions, the efficiencies of cut and heal tests were improved enormously. Herein, zinc acetate, DBTDL, and TBD were used as catalysts to evaluate the healing properties. It was observed that zinc acetate exhibited the best performance by the exchange reaction from view point of mechanical properties. It is postulated that thermal healing property could be implemented to ester-type TPU by blending with the phenoxy resin via efficient exchange reactions between ester, urethane, and hydroxy groups in the blends. 

## Figures and Tables

**Table 1:** Sample code and composition of the TPU/phenoxy resin blends.

Sample Codes	Blends		Catalysts		
	TPU	Phenoxy Resin	Zinc Acetate	DBTDL	TBD
	(g)	(g)	(wt%)	(wt%)	(wt%)
Ester TPU	100	0			
Ester TPU90/10	90	10			
Ester TPU80/20	80	20			
Ester TPU-zinc	100	0	0.1		
Ester TPU90/10-zinc	90	10	0.1		
Ester TPU80/20-zinc	80	20	0.1		
Ether TPU-zinc	100	0	0.1		
Ether TPU90/10-zinc	90	10	0.1		
Ether TPU80/20-zinc	80	20	0.1		
Ester TPU-DBTDL	100	0		0.1	
Ester TPU90/10-DBTDL	90	10		0.1	
Ester TPU80/20-DBTDL	80	20		0.1	
Ether TPU-DBTDL	100	0		0.1	
Ether TPU90/10-DBTDL	90	10		0.1	
Ether TPU80/20-DBTDL	80	20		0.1	
Ester TPU-TBD	100	0			0.1
Ester TPU90/10-TBD	90	10			0.1
Ester TPU80/20-TBD	80	20			0.1
Ether TPU-TBD	100	0			0.1
Ether TPU90/10-TBD	90	10			0.1
Ether TPU80/20-TBD	80	20			0.1

**Table 2:** Thermal, mechanical and dynamic mechanical properties of TPU/phenoxy resin blends.

Sample Codes	T_gs_ (°C)	T_m_ (°C)	T_c_ (°C)	Tensile Strength (MPa)	Elongation at Break (%)	Storage Modulus at 20 °C (MPa)
Ester TPU	−18	-	36	30.9 ± 1%	600 ± 1%	11.2
Ester TPU90/10	−12	155	44	27.8 ± 1.2%	615 ± 0.9%	37.2
Ester TPU80/20	−11	151	49	23.5 ± 1.1%	600 ± 0.8%	41.9
Ester TPU-zinc	−20	150	35	16 ± 1%	616 ± 0.8%	23.5
Ester TPU90/10-zinc	−12	145	38	19.7 ± 1.1%	615 ± 1%	28
Ester TPU80/20-zinc	−11	143	47	18.7 ± 1.2%	619 ± 1%	35.7
Ester TPU-DBTDL	−16	148	35	21.7 ± 1.5%	645 ± 1.3%	17.3
Ester TPU90/10-DBTDL	−11	145	42	16.6 ± 1.6%	583 ± 1.3%	22.8
Ester TPU80/20-DBTDL	−7	141	46	13.2 ± 1.5%	524 ± 1.3%	52.1
Ester TPU-TBD	−17	147	37	14.1 ± 1.5%	620 ± 1.2%	25.6
Ester TPU90/10-TBD	−13	144	40	9.2 ± 1.3%	615 ± 1%	20.3
Ester TPU80/20-TBD	−10	139	45	9 ± 1.4%	578 ± 1%	25.3
Ether TPU-zinc	−25	200	-	35 ± 0.9%	440 ± 1%	54.4
Ether TPU90/10-zinc	−22	197	-	32 ± 1%	360 ± 1.3%	133.8
Ether TPU80/20-zinc	−16	194	-	24 ± 1%	260 ± 1.5%	151.8
Ether TPU-DBTDL	−25	221	-	47 ± 1.2%	542 ± 1.6%	30.4
Ether TPU90/10-DBTDL	−18	200	-	28 ± 1%	396 ± 0.8%	120
Ether TPU80/20-DBTDL	−9	195	-	12 ± 1.1%	338 ± 1.3%	150.6
Ether TPU-TBD	−27	220	-	48.4 ± 1%	533 ± 1.2%	107.1
Ether TPU90/10-TBD	−21	201	-	44 ± 1.2%	520 ± 1.4%	118.5
Ether TPU80/20-TBD	−16	198	-	42 ± 1%	435 ± 1.2%	154.3

**Table 3:** Summary of the tensile properties and healing efficiency of TPU/phenoxy blends after heat treatment at 150 °C.

Sample	After Heat Treatment (without cut)		After Cut and Heal Test		Healing Efficiency (%)
	Tensile Strength (MPa)	Elongation at Break (%)	Tensile Strength (MPa)	Elongation at Break (%)	
Ester TPU	30.4	617	-	-	0
Ester TPU90/10	16.3	610	2.4	36	15
Ester TPU80/20	14.6	500	2.6	74	18
Ester TPU-zinc	7.1	600	-	-	0
Ester TPU90/10-zinc	15.8	509	14.1	478	89
Ester TPU80/20-zinc	8	434	7.7	418	96
Ester TPU-DBTDL	6.9	581	-	-	0
Ester TPU90/10-DBTDL	12.6	600	7	314	56
Ester TPU80/20-DBTDL	8.6	600	7	570	82
Ester TPU-TBD	4	200	-	-	0
Ester TPU90/10-TBD	4.3	184	4.1	150	95
Ester TPU80/20-TBD	3.9	210	3.9	165	100

## Supplementary Materials

The Supplementary Materials are available online at https://www.mdpi.com/2073-4360/12/5/1011/s1. Scheme S1: Schematics of self-healing process. Table S1: Molecular weight of TPU and phenoxy resin. Figure S1: (a) FT-IR spectra and (b) ^1^H-NMR spectra of EE and phenoxy, respectively. Figure S2: TGA thermograms of ester-type TPU/phenoxy blends, (a) ester-tpe TPU/phenoxy resin blends without catalyst, (b) ester TPU blends under catalyst. Figure S3: DMA curves of ether-type TPU/phenoxy blends: (a) Storage modulus for the ether-type TPU blends with zinc acetate as catalyst; (b) Storage modulus for the ether-type TPU blends with DBTDL as catalyst; (c) Storage modulus for the ether-type TPU blends with TBD as catalyst. Figure S4: Tan Delta curves of ester-type TPU/phenoxy blends: (a) Ester-type TPU blends without catalyst; (b) Ester-type TPU blends with zinc acetate as catalyst; (c) Ester-type TPU blends with DBTDL as catalyst; (d) Ester-type TPU blends with TBD as catalyst. Figure S5: Tan Delta curves of ether TPU/phenoxy blends: (a) Ether-type TPU blends with zinc acetate as catalyst; (b) Ether-type TPU blends with DBTDL as catalyst; (c) Ether-type TPU blends with TBD as catalyst.
