# Supplementary material for: Self-Healing and Mechanical Properties of Thermoplastic Polyurethane/Eugenol-Based Phenoxy Resin Blends via Exchange Reactions
Source: Polymers (Basel). 2020 Apr 28;12(5):1011. doi: 10.3390/polym12051011 (PMC7284341; doi:10.3390/polym12051011)
Supplement: Supplementary file 1 [file polymers-12-01011-s001.pdf]

Supporting information

Self-healing and mechanical properties of thermoplastic polyurethane/eugenol-based phenoxy resin blends via exchange reactions

Jing-Yu Liang [1†], Se-Ra Shin[2†], Soo-Hyoung Lee[1*], and Dai-Soo Lee [3,*]

[1] Department of Semiconductor and Chemical Engineering, Chonbuk National University, 567 Baekje-daero, Deokjin-gu, Jeonju 54896, Korea; liangjy@naver.com (J.-Y.L.)

[2] Research Institute of Jungwoo Fine Chemical, 68-3 Seogam-ro 1-gil, Iksan, 54586, Korea: srshin89@jbnu.ac.kr (S.-R.S.)

[3] Research Institute of Future Energy, Chonbuk National University, 567 Baekje-daero, Deokjini-gu, Jeonju 54896, Korea

* Correspondence: shlee66@jbnu.ac.kr (S.-H.L.); daisoolee@jbnu.ac.kr(D.-S.L); Tel.: +82-63-270-2435(S.H.L); +82-10-6660-7693(D.S.L)

† Contributed equally to this work.

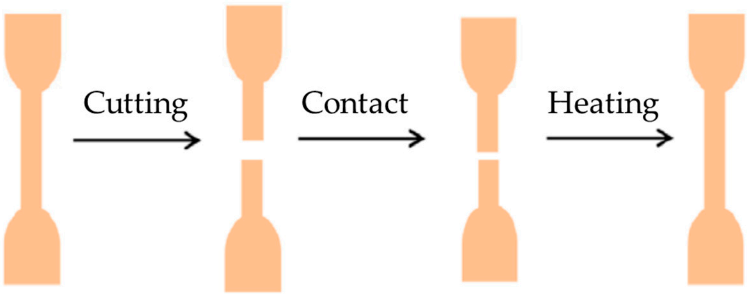

Scheme S1. Schematics of self-healing process.

Table S1. Average weights of TPUs and phenoxy resin determined by GPC*.

Sample	Average Molecular Weight			
	M_n	M_w	M_z	M_w/M_n
Ester-type TPU	45,000	83,000	130000	1.83
Ether-type TPU	56,000	120,000	240000	2.17
Phenoxy resin	2,400	4,000	7400	1.74

* Average molecular weights of TPU and phenoxy resin were determined by GPC (Agilent 1200S; Agilent) employing a refractive index detector (Optilab rEX; Wyatt). Samples were dissolved in DMF/THF (1:1 wt./wt.), and polystyrene standards were used for universal calibration.

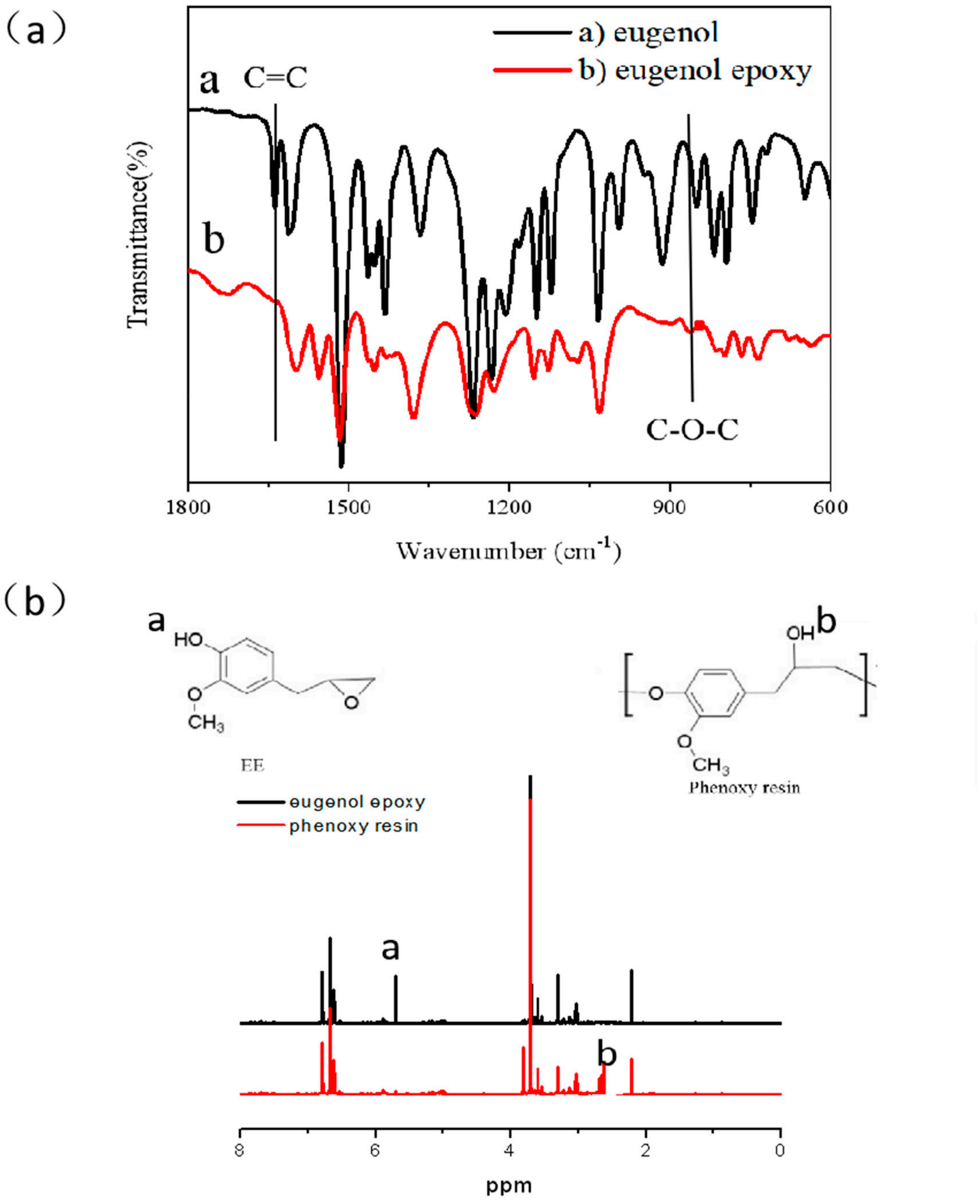

Figure S1. FTIR spectra **(a)** and ^{1}H NMR spectra **(b)** of EE and phenoxy.

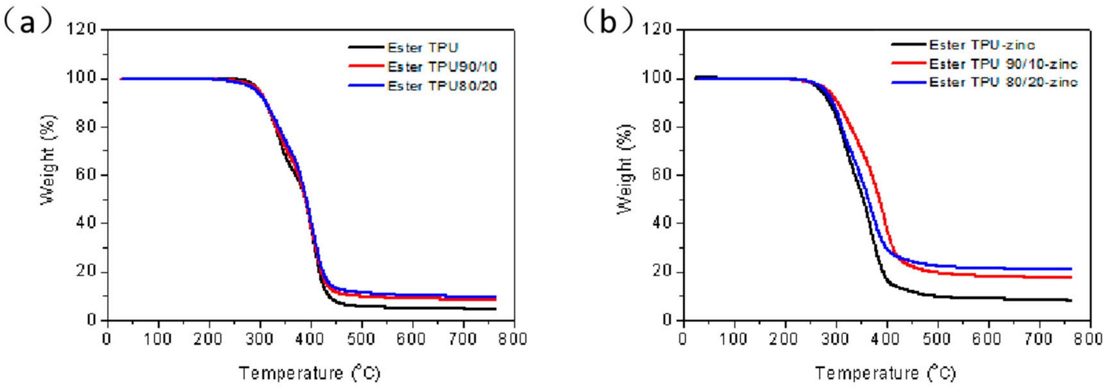

Figure S2. TGA thermograms of ester-type TPU/phenoxy blends without catalyst (a), ester-type TPU/phenoxy blends in the presence of catalyst, zinc acetate.

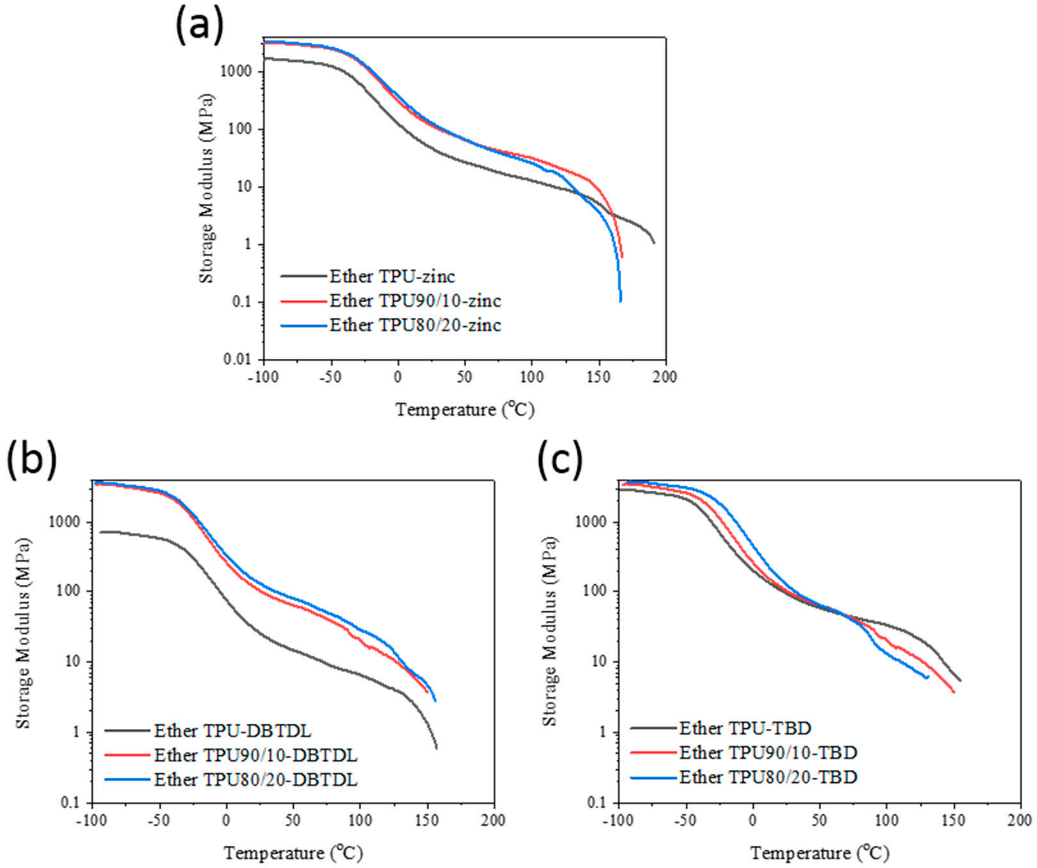

Figure S3. DMA curves of ether TPU/phenoxy blends, (a) Storage modulus for the ether-type TPU blends with zinc acetate as catalyst, (b) Storage modulus for the ester-type TPU blends with DBTDL as catalyst, (c) Storage modulus for the ether-type TPU blends with TBD as catalyst.

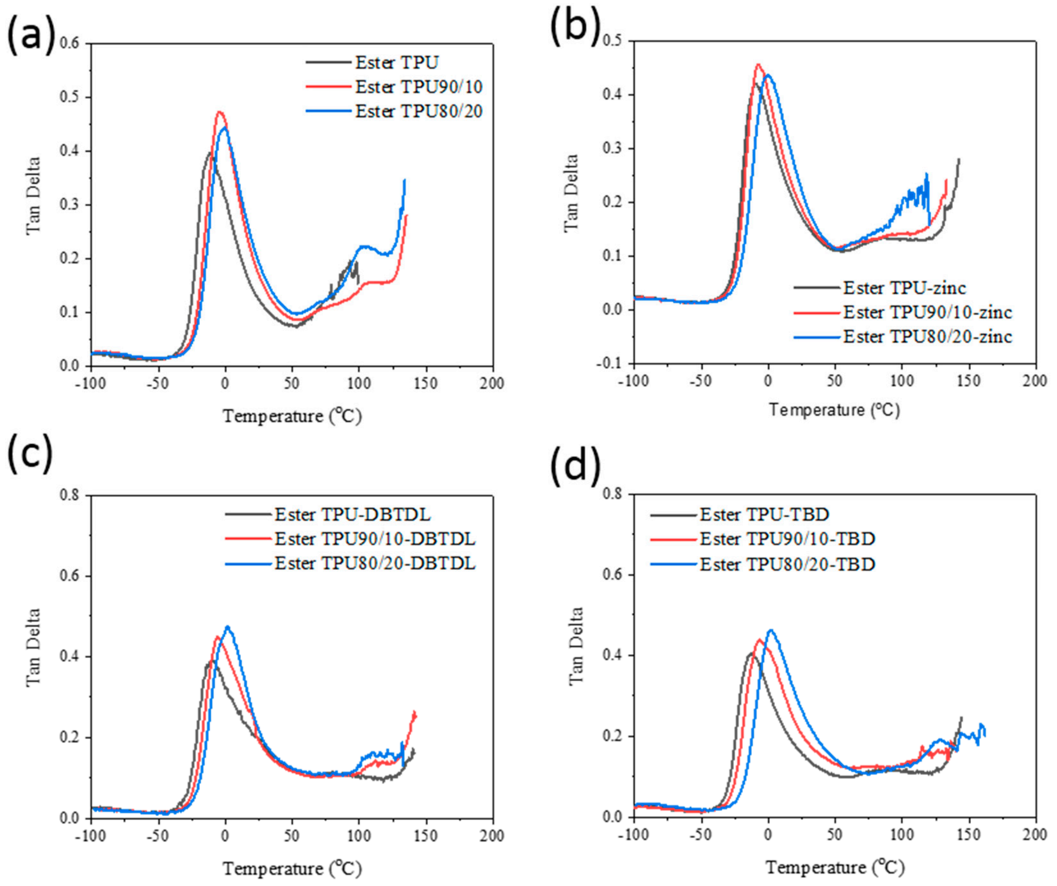

Figure S4. Tan Delta curves of ester TPU/phenoxy blends, (a) Ester-type TPU blends without catalyst, (b) Ester-type TPU blends with zinc acetate as catalyst, (c) Ester-type TPU blends with DBTDL as catalyst, (d) Ester TPU blends with TBD as catalyst.

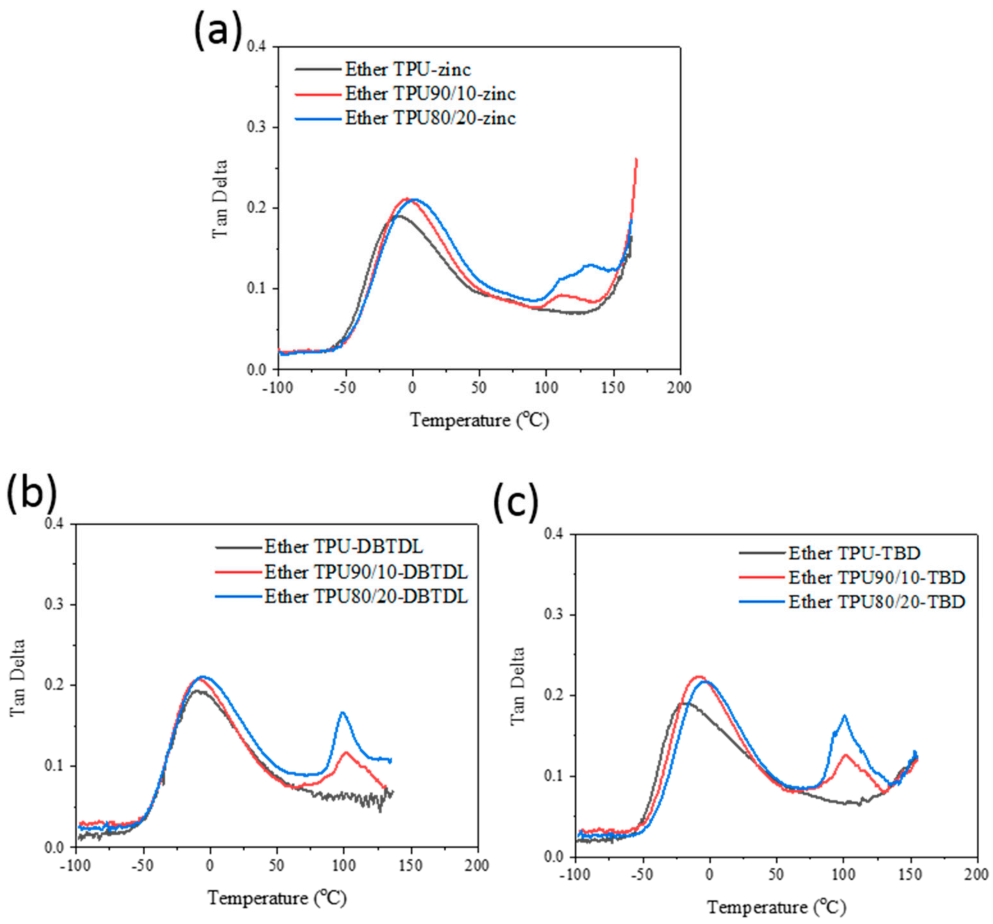

Figure S5. Tan Delta curves of ether-type TPU/phenoxy blends, (a) Ether-type TPU blends with zinc acetat e as catalyst, (b) Ether-type TPU blends with DBTDL as catalyst, (c) Ether-type TPU blends with TBD as c atalyst.
